# Supplementary material for: GluR2Q and GluR2R AMPA Subunits are not Targets of lypd2 Interaction
Source: PLoS One. 2022 Nov 28;17(11):e0278278. doi: 10.1371/journal.pone.0278278 (PMC9704558; doi:10.1371/journal.pone.0278278)

Fig1A

Images captured using BioRad gel imager, EtBr setting; “X” refers to lanes containing other samples not relevant to this publication

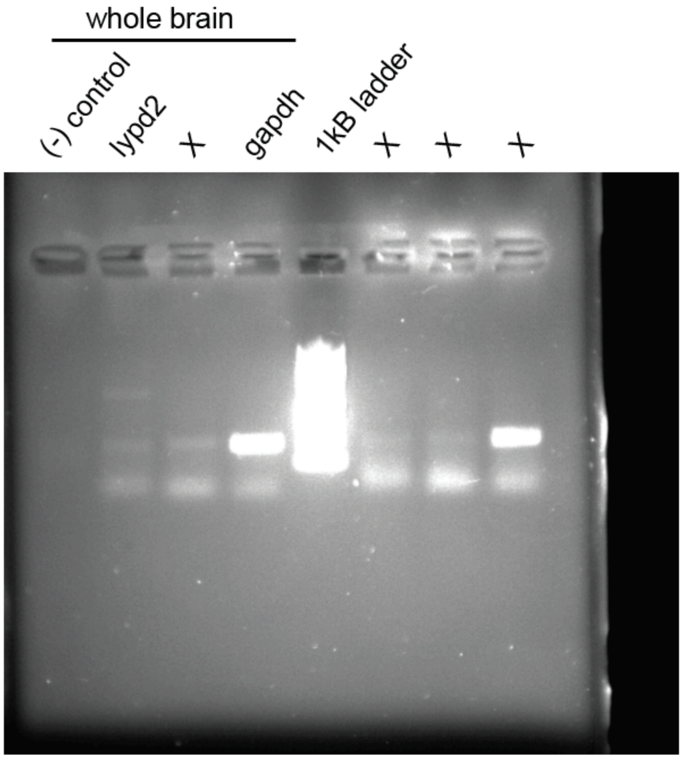

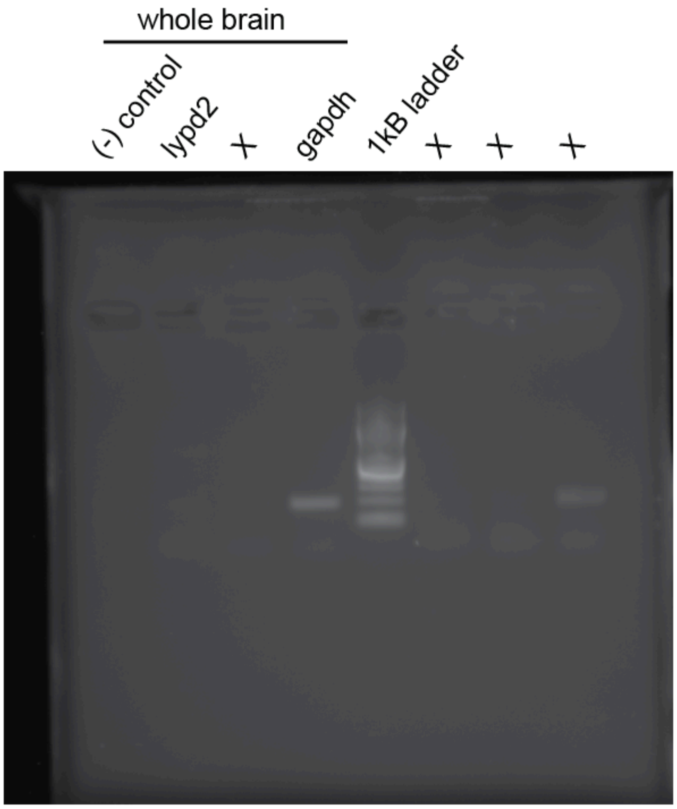

Both images above are of the same gel at different exposures: the image at the right was used to verify the correct size of the PCR product.

Fig1A
Images captured using BioRad gel imager, EtBr setting; “X” refers to lanes containing other samples not relevant to this publication

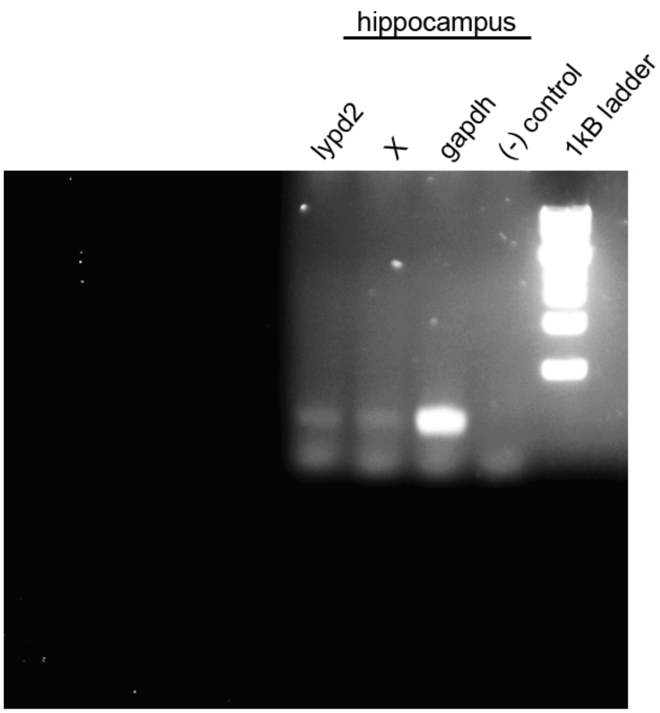

Fig1B
GE Lifesciences WB Imager

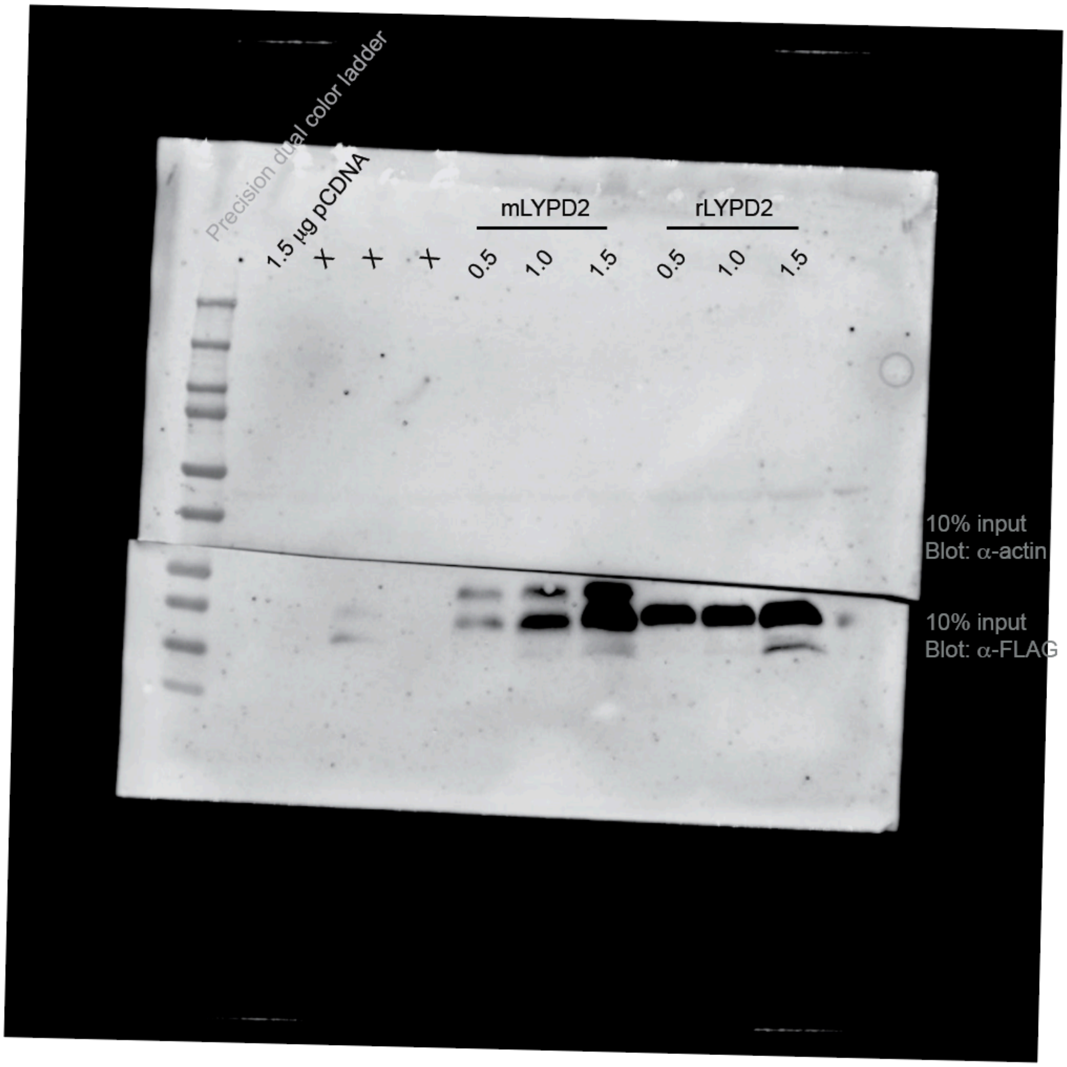

Figure 2A: GE Lifesciences WB Imager. “X” refers to lanes containing other samples not relevant to this publication

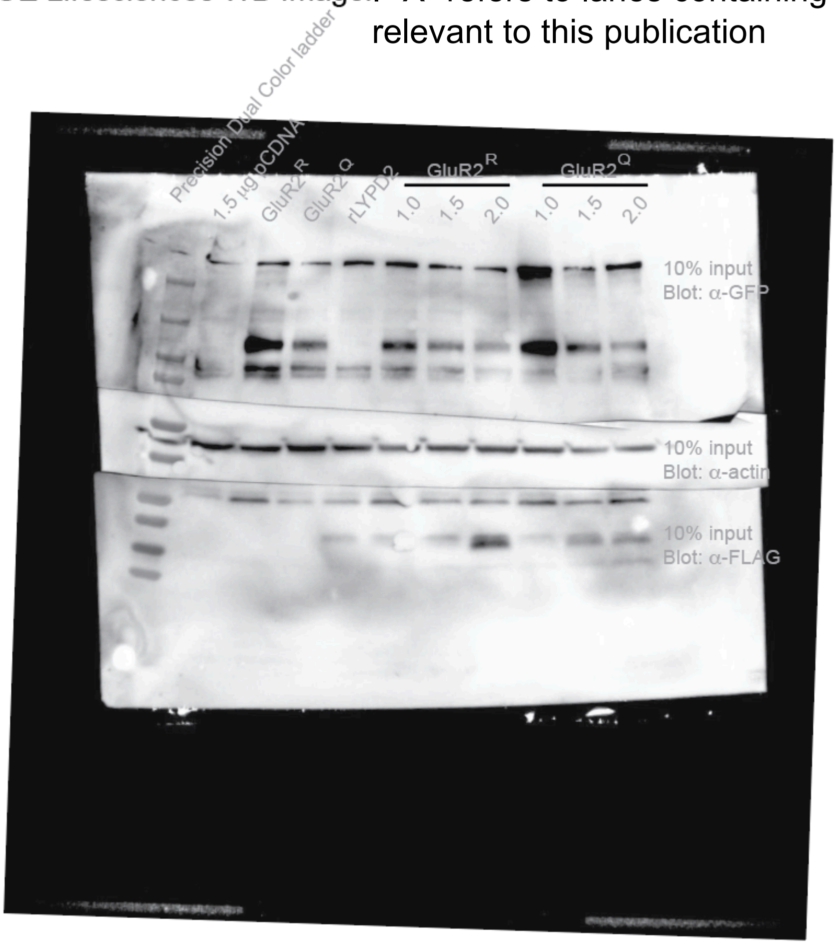

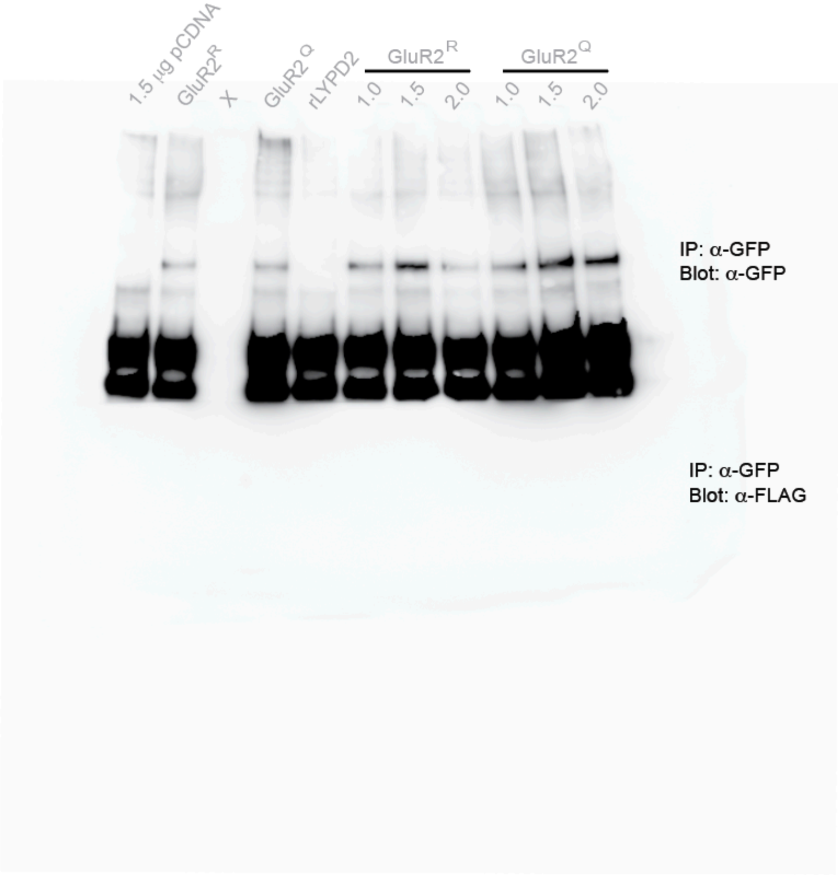

Figure 2B: GE Lifesciences WB Imager. "X" refers to lanes containing other samples not relevant to this publication
4m exposure

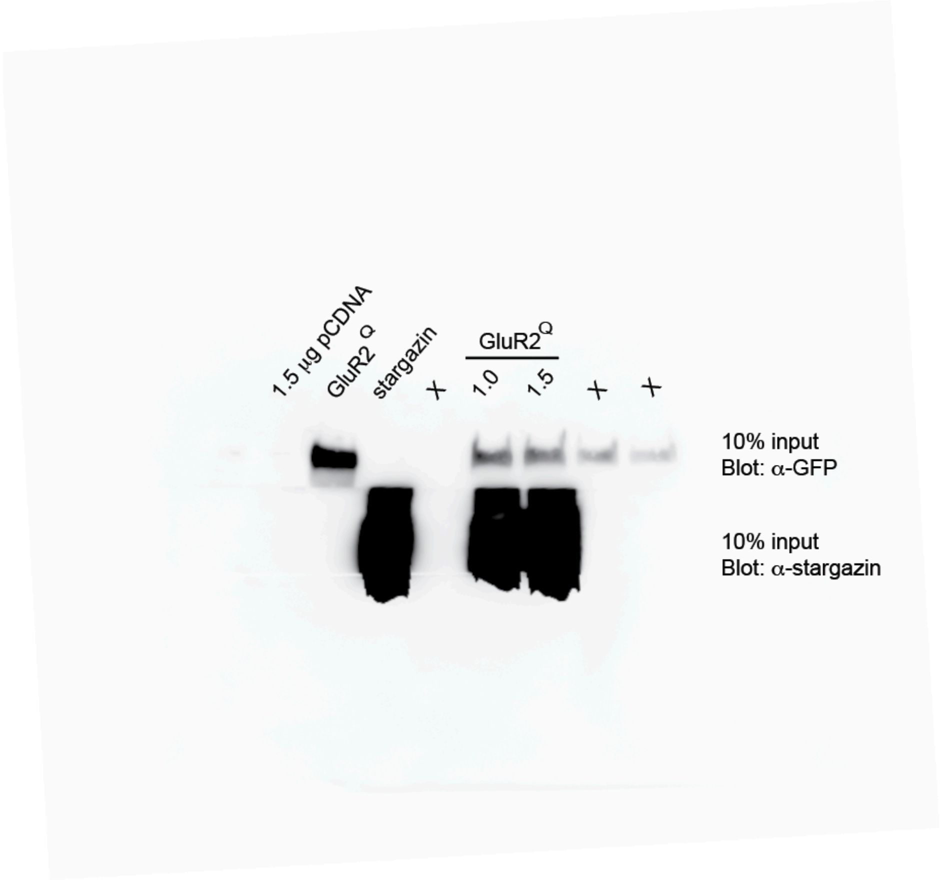

Figure 2B: GE Lifesciences WB Imager
1m exposure

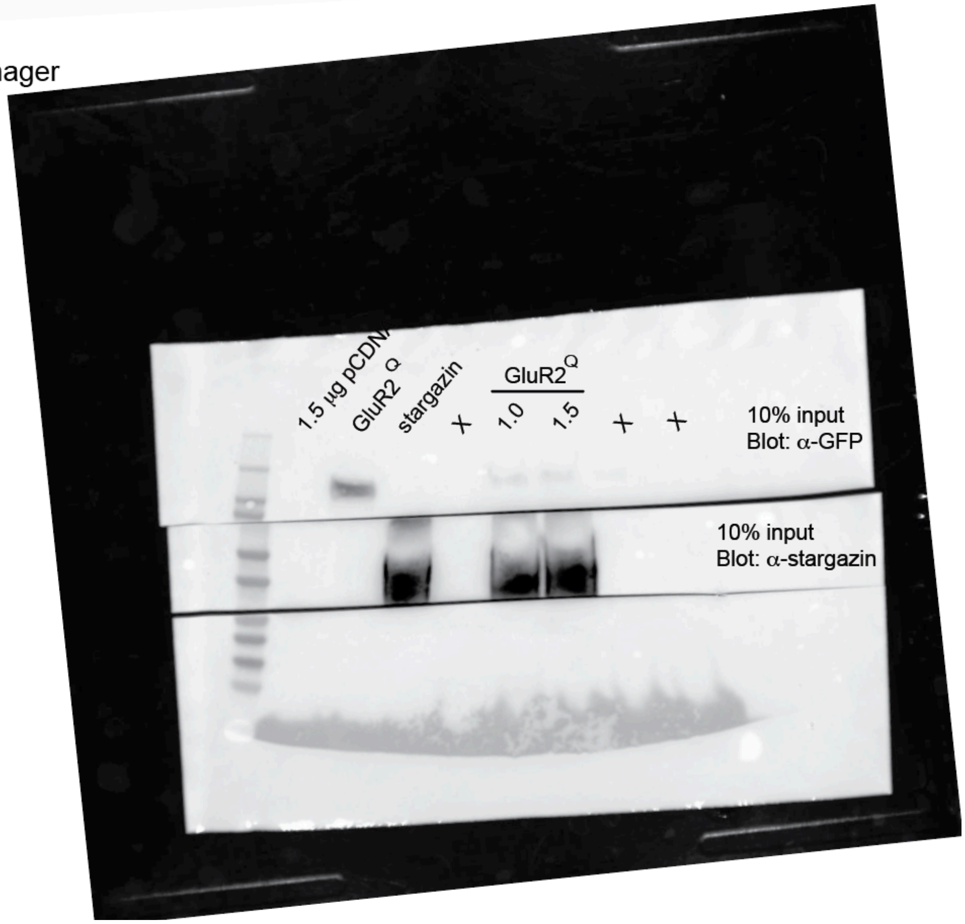

Figure 2B: GE Lifesciences WB Imager. "X" refers to lanes containing other samples not relevant to this publication
1m exposure

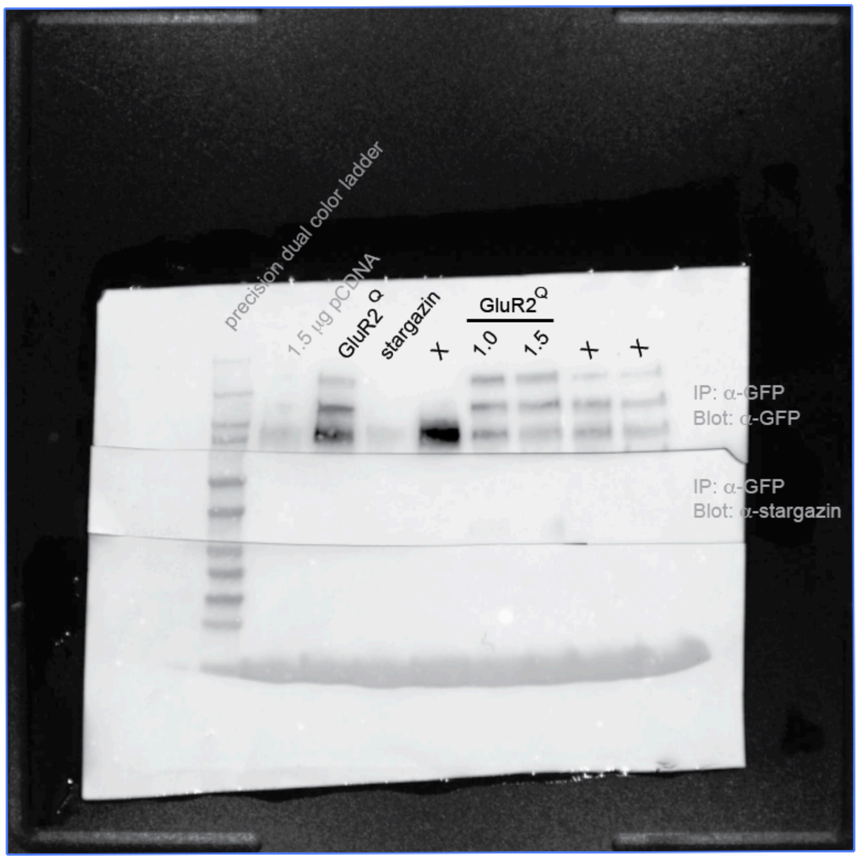

Figure 2B: GE Lifesciences WB Imager
4m exposure

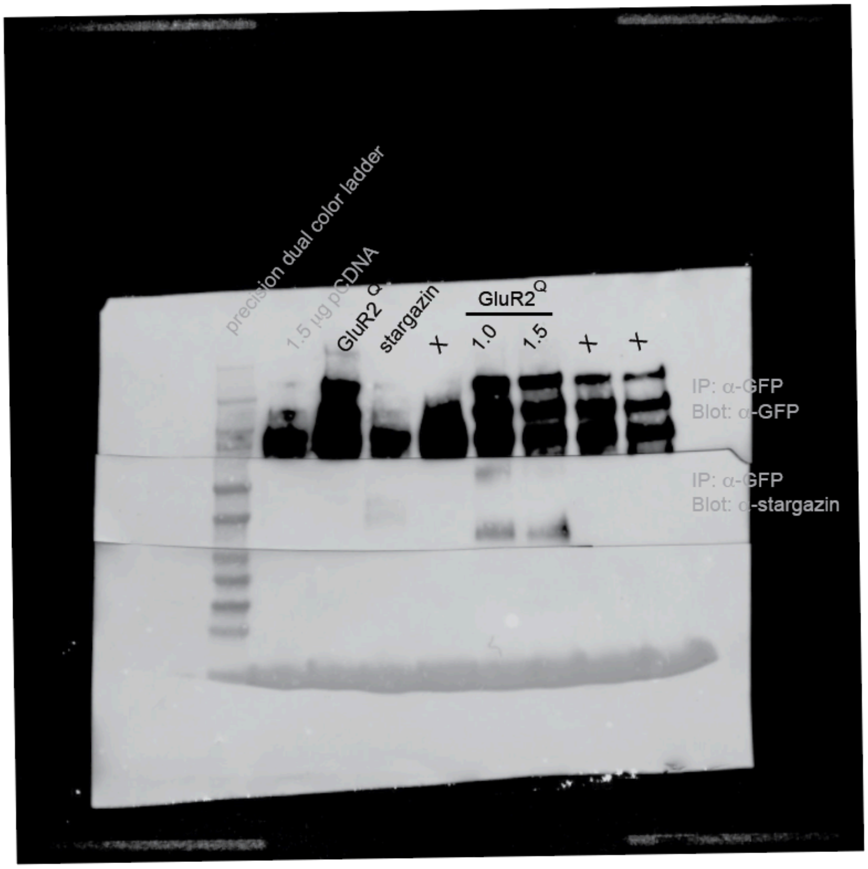

Figure 2C: GE Lifesciences WB Imager. “X” refers to lanes containing other samples not relevant to this publication

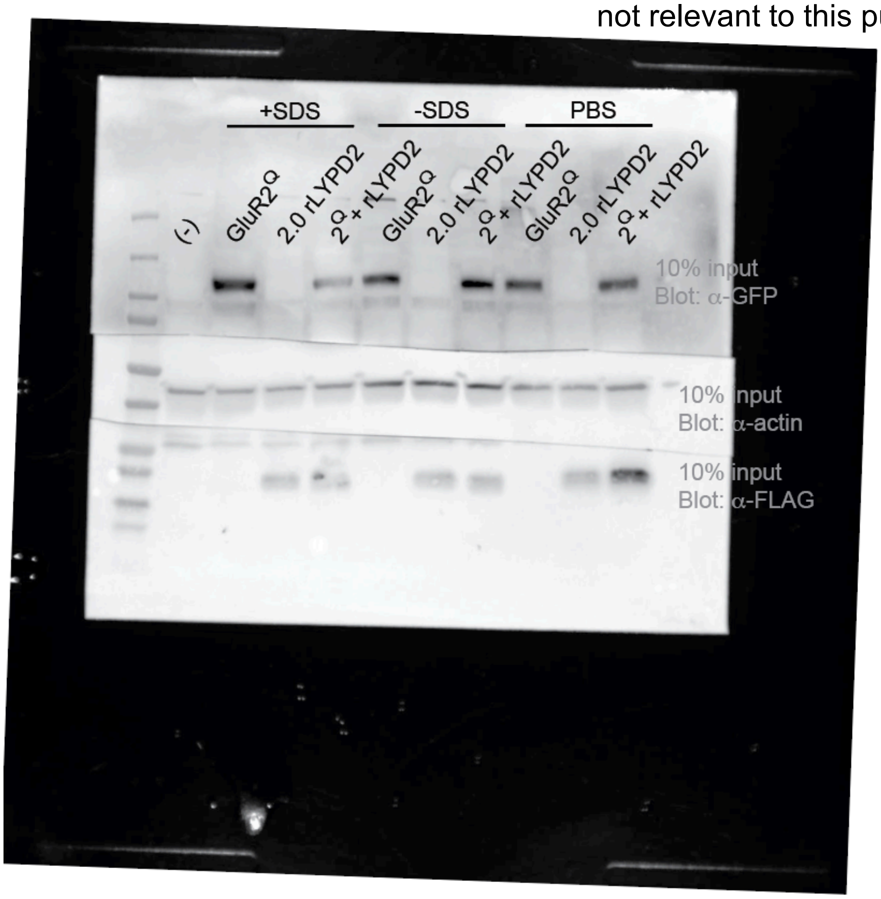

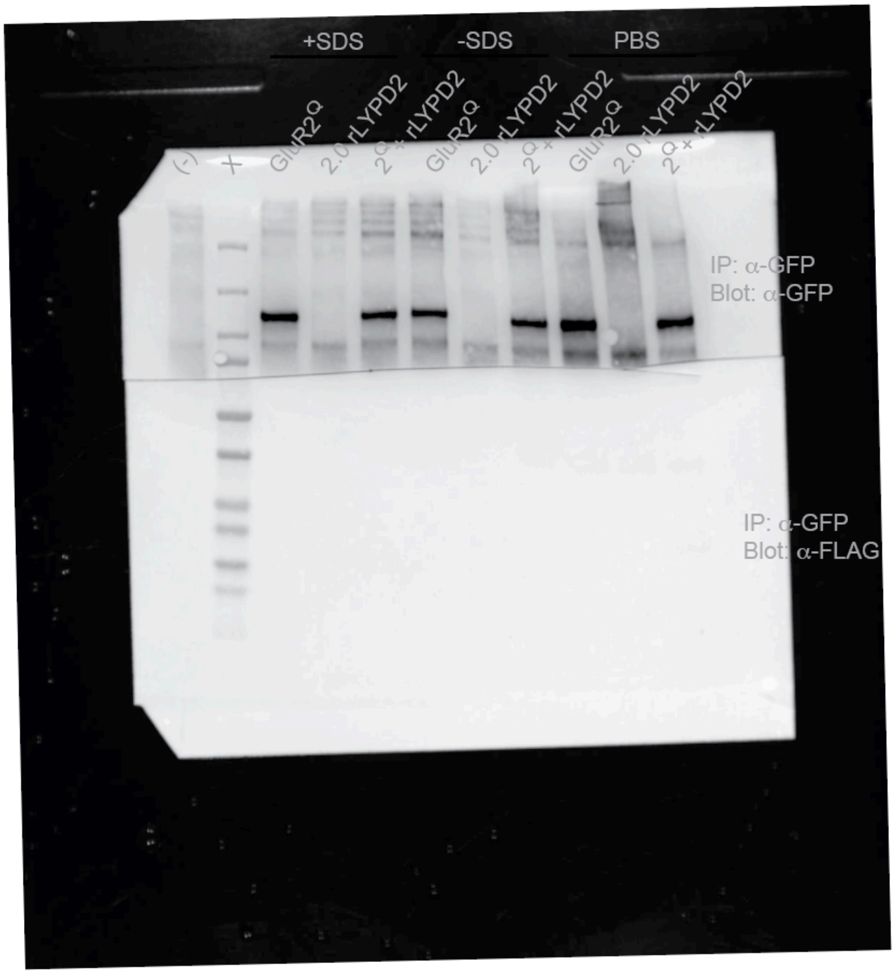

Supplement: S1 Raw images — (PDF) [file pone.0278278.s001.pdf]
