# Supplementary material for: GluR2Q and GluR2R AMPA Subunits are not Targets of lypd2 Interaction
Source: PLoS One. 2022 Nov 28;17(11):e0278278. doi: 10.1371/journal.pone.0278278 (PMC9704558; doi:10.1371/journal.pone.0278278)
Supplement: Attachment — Submitted filename: Response to Reviewers.docx [file pone.0278278.s002.docx]

August 26th, 2022

Dear Dr. Silman,

We thank you and the two reviewers for their time and effort in evaluating our manuscript, “GluR2^Q^ and GluR2^R^ AMPA Subunits are not Targets of lypd2 Interaction” (**PONE-D-22-12127**). We are highly appreciative of the detailed comments provided and were especially gratified to see that Reviewer #2 found the negative results to be “worth noting.”

We have addressed the comments and requests for revisions from the expert reviewers, including an additional figure and text to clarify points of confusion (see below). In doing so, we believe we have significantly improved and strengthened our manuscript. In the following, we provide an itemized list of changes we have made in addressing the provided comments.

Thank you again for your feedback and assistance with our manuscript. We look forward to continuing to work with you, and hope to hear from you soon.

Sincerely,

Rou-Jia Sung, Ph.D.

**Response to Reviewers**

**Reviewer 1:**

*“The task formulated by the authors - to detect novel receptors with which may interact the proteins of the Ly6/uPAR family - is well justified. They correctly started by emphasizing the well-known common three-finger folding of these proteins and of the three-finger alpha-neurotoxins from the snake venoms. However, they did clearly mention that there are two classes of the Ly6/uPAR proteins - secreted like SLURP-1 and membrane-bound by the GPI anchor like Lynx1. They gave the reasons why they have chosen lypd2 (because there are data on its colocalization with the AMPA receptors) but did not mention that lypd 2 is membrane-bound.”*

We thank the reviewer for this careful attention to detail and have noted on line 126 that lypd2 is predicted to be a GPI-anchored protein.

*“The authors made several mistakes in the introduction concerning snake venom neurotoxins:
line 49: major binding site of the snake venom alpha-neurotoxins is the orthosteric binding site (where agonists are bound), but not the allosteric sites (where mainly other allosteric positive or negative modulators are attached),”*

We thank the reviewer for noting this error and have clarified on line 55 that alpha-neurotoxins primarily exert their effects through the orthosteric site, not the allosteric site.

*“line 52: give references. line 53: alpha -bungarotoxin does not inhibit alpha4beta2 receptors, and instead of alpha7beta1 should be written "alpha7beta2.”*

We have included additional references on line 58 on the earliest work studying lynx1 modulation of nicotinic acetylcholine receptors (references 19 and 20). We have corrected and clarified the effects of bungarotoxin on inhibition of homopentameric alpha7 receptors while lynx1 and lynx2 have modulatory effects on alpha7 and alpha4beta2 receptors on lines 59-61, and included an additional reference (reference 22).

*“line 20 and through the whole text authors use the term "ly6 proteins" , although most often in the literature are used Ly6 or Ly6/uPAR proteins.”*

We have gone through the manuscript and replaced “ly6 proteins” or “ly6” with “Ly6 proteins” and “Ly6.”

**Reviewer 2:**

*“Overall, there is very little about where ly6-AMPAR interaction is expected. If the experiments are limited for whatever reason, it does not make much sense to look for possible interactions of lypd2 with Q or R variants of GluA2. If the experiments are limited for whatever reason, it does not make much sense to look for possible interactions of lypd2 with Q or R variants of GluA2. It would make more sense to test other AMPAR subunits (A1, A3, A4, including heteromers) or complexes of AMPARs with auxiliary subunits. That would make the study more informative with the same level of experimentation. The reason for this is predominantly extracellular location of ly6 proteins: they are either secreted or GPI-anchored to the outer leaflet of the plasma membrane whereas the Q/R editing site is in the pore of AMPARs, i.e. in the transmembrane region. It is not clear how the two could interact. Of course, the only way to rule this out is by experiments, however, if experiments are limited as it seems to be the case here, then it would make more sense to focus on variation in extracellular parts of AMPARs (provided by different AMPAR core subunits and complexes of AMPARs with auxiliary subunits) as these are more likely sites of interaction with lypd2 (ly6-related alpha-bungarotoxin also interacts with extracellular domains of acetylcholine receptors).”*

We thank the reviewer for this feedback. We agree that it does not make sense to look for interactions of lypd2 with the amino acids at position 607, representing the Q/R editing site on GluR2, and it was not our original intention to identify or propose direct interactions between lypd2 and the Q/R amino acid site. To help clarify this confusion, we have:

1) Included Figure 1 (lines 117-123) at the reviewer’s suggestion below to highlight where we expect Ly6-GluR2 receptor interactions to occur at the structural level. In this model, based on the scale of each molecule relative to each other, it is likely that a Ly6 protein could make contact with the ligand binding domain and/or the linker between the ligand binding domain and the transmembrane domain.
2) Striven to clarify our logic with regards to why we chose to focus on the Q and R variants of the GlRA2 homomer in our study in lines 131-166. Our interest in these isoforms was on the basis of literature indicating isoform-specific differences in interactions and regulations with small regulatory proteins such as the type-II TARPs γ-5 and γ-7.
3) We have also expanded the discussion (lines 322-328) to acknowledge that there are other AMPAR variations in terms of subunit composition and editing events that we did not explore that could also be possibilities for interaction.

*“The authors even do pull down Stargazin and AMPAR complexes, but for some reason do not test these for the possible presence of lypd2.”*

Given that AMPAR receptors have been shown to participate in dimeric complexes (detected as an interaction between the receptor and a single auxiliary protein of interest using a transfected cell culture system absent other auxiliary proteins), our goal in this study was to delineate the possibility of an interaction between Ly6 proteins and AMPARs using a similar approach. Consequently, the purpose of the stargazin pulldown was to serve as a positive control for our pulldown conditions, not for the purposes of detecting a trimeric complex. We thank the reviewer for this feedback, however, and we highlight in our expanded discussion in lines 327-336 that it is certainly possible that there could be trimeric complexes between AMPAR, Ly6 proteins, and these auxiliary proteins and indicate that this could be a source of future exploration.

*“The authors correctly note rich repertoire of AMPAR auxiliary subunits, however: Line 76: please note that Stargazin is a TARP.”*

We thank the reviewer for this careful attention to detail and have noted on line 97 that stargazin is a TARP.

*“Lines 77-80 – it is not clear to me at all how AMPAR auxiliary proteins share cellular location and molecular architecture with ly6 proteins. Listed AMPAR auxiliary proteins are all transmembrane proteins whereas ly6 proteins are secreted or GPI-anchored to the outer leaflet of the plasma membrane. The authors state that AMPAR auxiliary subunits are heavily stabilized by disulphide bonds which is not the case. They provide a 2010 reference to support this statement, however, no structures of auxiliary subunits were available then. Meanwhile, structures of TARPs y2 (Stargazin), y8 and y5 came out as well as of CNIHs 2 and 3 which do not show extensive disulphide linking.”*

We thank the reviewer for this feedback, and have updated both our text in the manuscript and our references accordingly. The new structural information available on AMPAR-auxiliary protein structures is very exciting, and indeed shifts our perspective on the similarities between these auxiliary proteins and Ly6s—as we have modified in the text, it is evident that the similarities are less in molecular structure and more in the size and placement of these small regulatory proteins. Both Ly6s, TARPS, and CNIHs are located in close proximity to the plasma membrane, which limits their contact points to largely the ligand binding domain and/or the linker region between the ligand binding domain and the transmembrane domain of the AMPAR. We have clarified our language in the introduction to reflect this in lines 99-111 and 161-163.

*“Please, also note that AMPARs in the brain are almost always associated with auxiliary subunits (see e.g. Yu et al, Nature, 2021;* [*https://doi.org/10.1038/s41586-021-03540-0*](https://doi.org/10.1038/s41586-021-03540-0)*), which means that if lypd2 was interacting with native AMPARs in hippocampus, it would have to do so with AMPARs complexed with auxiliary subunits (y8 and CNIH2). RT-PCR is used to show expression of lypd2 in hippocampus and although AMPARs are also expressed in hippocampus, this does not strictly mean co-localization. This should be at least acknowledged in the discussion.”*

We thank the reviewer for this thoughtful feedback. We have expanded the discussion in lines 322-336 to acknowledge the questions left unexplored in our current study and to consider these possibilities as avenues for future exploration.

*“I don’t think the ratio of Stargazin – A2 DNA used in transfections is mentioned anywhere.”*

We have updated the figure legend on line 303-304 for Figure 3 with the ratio of transfected DNAs. *“Maybe I missed it, but it is not mentioned anywhere how many times Western Blots or imaging experiments were replicated.”*

All figures for Westerns and imaging experiments are representative images from at least three replicates; we have noted this in lines 225-226 and 247-248 in the methods.

*“The figures are quite low in resolution.”*

The figures were created using digital image files saved from our GE ImageQuant Western Blot Imager. We note the reviewer’s feedback, but unfortunately are only able to work with the image resolution from the saved images. We will examine the settings on our instrument to see if there is the possibility of improving the image resolution for future work.

*“A panel or a figure to introduce the topic would be very helpful, e.g. AMPAR structure (including Q/R editing site) and a model of ly6 protein in the same membrane (UniProt entry for lypd2 provides alphafold structure which might be useful).”*

We thank the reviewer for their feedback and have created Figure 1 to show a juxtaposition of structural data for GluR2 and lynx1 as well as the Alphafold model of lypd2. Since the structure of lynx1 has been experimentally determined, we chose to use lynx1 in panel A to maintain accuracy in scale and structural detail; however, as shown in panel B it is likely that lypd2 shares significant structural homology to lynx1. We have introduced and explained this model in the text as well, in lines 117-123 and 155-163.

*“Raw images of gels relating to Fig. 1A are quite over-exposed. The amount of loaded ladder should be reduced to make it readable. Also, please explain what “X” stands for here.”*

“X” refers lanes containing other samples not relevant to this publication and cropped out of the final figures in the manuscript; we have appended the labeling associated with our raw image files to reflect this annotation. We have also included an additional exposure of the gel for the whole brain sample in which the ladder was not overexposed to demonstrate the correct size of the expected PCR products.

*“Figure 1B – actin bands are very faint. Again, there seem to be some bands in a well marked “X”.”*

We note the reviewer’s critique here, and acknowledge that the actin bands are very faint. However, we do not have the ability to reexpose that specific blot as the physical blot itself was discarded after those initial exposures. The lanes marked X have been previously described in the legend for Fig 1A, and apply to all subsequent figures.

*“Figure 1C – why is over-expressed lypd2 visible as puncta? Is it aggregating?”*

GPI anchored Ly6 proteins have been previously observed to form puncta, possibly due to localization to microdomains in the plasma membrane. It is thought that these puncta represent functional organization of the Ly6 proteins at the plasma membrane (in contrast to non-functional aggregates), although additional details remain unknown. We have included additional references in the text (line 261).

*“Discussion is very limited and should be extendend (e.g. by acknowledging that the performed experiments do not entirely exclude interaction between lydp2 and AMPARs as other core subunits have not been tested nor complexes of AMPARs with auxiliary subunits).”*

We thank the reviewer for this feedback and have expanded the discussion in lines 323-337 to highlight the these potential avenues of future exploration with regards to other AMPARs containing other core subunits as well as the possibility of trimeric complexes between AMPARs, auxiliary subunits, and lypd2.

*“Some typos:
Abstract: “alpha” shows up as “a”
Line 67 – delete “of”
Line 100 – was instead of were?
Line 120 – degree sign missing after 37
Line 122 – 2 mM”*

We thank the reviewer for such a close reading of the text, and these typos have been addressed in the revised manuscript text.
