# Supplementary material for: GluR2Q and GluR2R AMPA Subunits are not Targets of lypd2 Interaction
Source: PLoS One. 2022 Nov 28;17(11):e0278278. doi: 10.1371/journal.pone.0278278 (PMC9704558; doi:10.1371/journal.pone.0278278)
Supplement: Attachment — Submitted filename: Response to Reviewers.docx [file pone.0278278.s003.docx]

October 25^th^, 2022

Dear Dr. Silman,

We thank you and the two reviewers for their time and effort in evaluating our manuscript, “GluR2^Q^ and GluR2^R^ AMPA Subunits are not Targets of lypd2 Interaction” (PONE-D-22-12127R1). We appreciate the time and energy both you and the reviewers have spent on our manuscript; in particular, we remain gratified that Reviewer #2 considers our study an interesting question that provides a result to the community.

We have endeavored to address the comments and requests for revision from Reviewer #2. In particular, I leverage my expertise as a structural biologist and protein biochemist in conjunction with additional references from the literature to clarify the logic behind which interactions we hypothesized between GluR2 and lypd2. We acknowledge that there may still be points of disagreement, but we hope this additional context and explanation provides insight into our reasoning and helps address points of confusion. In the following, we provide an itemized list of changes we have made in addressing the provided comments.

Thank you again for your feedback and assistance with our manuscript. We look forward to continuing to work with you, and hope to hear from you soon.

Sincerely,

Rou-Jia Sung, Ph.D.

**Response to Reviewers**

**Reviewer #1:** 
*I am glad that my critical comments were taken into account and the required changes were introduced into the revised version.*

**Reviewer #2:** 
*I thank the authors for going through all my points and in particular for including a new figure (Fig. 1) as I know this can take time.*

We thank both reviewers for their attention to our manuscript, and we feel the manuscript has been significantly improved on the basis of their feedback.

**Reviewer #2**

*“Yes, they are both (Ly6 and auxiliary proteins) associated with membrane, but all AMPAR auxiliary proteins are transmembrane proteins and interact fairly tightly with AMPAR transmembrane regions. Hence, their interactions with AMPARs can be expected to be sensitive to Q/R editing site in the pore.* *Ly6 proteins are anchored to the membrane, but are not transmembrane proteins. Their interaction points are most likely to be with the extracellular domains of AMPARs as shown in Fig. 1.”*

We respectfully disagree with the expectation that the interaction between the transmembrane domains of AMPARs and auxiliary proteins are the molecular basis for their regulation of AMPAR function. The interaction between transmembrane domains may be important for complex formation and stability, but prior work mapping the functional interface between TARPs and AMPARs have shown that 1) the extracellular loops of TARPs mediate their functional effects on AMPARs and 2) the regions important for mediating these effects on AMPARs include the linker region between the N-terminal domain (NTD) and ligand binding domain (LBD) for γ-2 and the linker region between the LBD and the transmembrane domain (TMD) for γ-8 ^1,2,3^. Ly6 proteins, despite not having a transmembrane domain, are positioned well to interact with any of these linker regions to potentially mediate functional impacts in a similar mechanism as the extracellular loops of TARPs.

^1^Ben-Yaacov A, Gillor M, Haham T, Parsai A, Qneibi M, Stern-Bach Y. Molecular Mechanism of AMPA Receptor Modulation by TARP/Stargazin. Neuron. 2017 Mar;93(5):1126-1137.e4.

^2^Cais O, Herguedas B, Krol K, Cull-Candy SG, Farrant M, Greger IH. Mapping the Interaction Sites between AMPA Receptors and TARPs Reveals a Role for the Receptor N-Terminal Domain in Channel Gating. Cell Rep. 2014 Oct;9(2):728–40.

^3^Herguedas B, Watson JF, Ho H, Cais O, García-Nafría J, Greger IH. Architecture of the heteromeric GluA1/2 AMPA receptor in complex with the auxiliary subunit TARP γ8. Science. 2019 Apr 26;364(6438).

We have added additional language in the introduction (lines 81-87 and 124-128) to 1) clarify that auxiliary protein mediated regulation of AMPARs are likely to be mediated through contacts between the TMD-LBD or LBD-NBD linkers, not direct contact with the Q/R site and 2) include the additional citations above.

*“So why not test then other AMPARs subunits and/or variants which differ in the extracellular region and not just in the pore region?”*

Our experiments do not rule out the possibility of other domains on the AMPAR as playing a role in the interaction. We have included language in the discussion (lines 292-293) to clarify this; we describe the logic of our reasoning in a response to a separate reviewer comment further below.

“*Yes, Q/R editing site is very important for kinetics, trafficking, assembly etc. but majority of these come from the fact that this residue is in the pore and directly determines calcium permeability and pore properties.”*

The biochemical mechanism behind the role of the Q/R editing site in determining calcium permeability and pore properties is quite clear and has been previously established; however, this only encapsulates direct effects post-assembly once the full pore has been established within a correctly folded and assembled AMPAR tetramer. It has also been shown that the editing at this site plays a critical role in the steps of AMPAR maturation prior to complete assembly. Greger et al^4^ shows changes in export kinetics from the ER between Q and R forms, indicating that the change in amino acid at this position has potential impacts on AMPAR function at a stage in its biogenesis where pore formation may not have occurred (i.e during protein folding and/or assembly). Moreover, Cais et al^2^ observed Q/R isoform-specific differences in association between γ-2 and AMPARs lacking an NTD (decreased co-immunoprecipitation with GluR2^R^ vs GluR2^Q^), suggesting that the effects of the Q/R editing site extend beyond direct impacts to the pore.

^4^Greger IH, Khatri L, Ziff EB. RNA Editing at Arg607 Controls AMPA Receptor Exit from the Endoplasmic Reticulum. Neuron. 2002 May;34(5):759–72.

Consequently, the role of the Q/R site in AMPAR function extends beyond its direct function in the pore to include indirect effects on AMPAR biogenesis as well. We have added language to 1) distinguish the effects of the Q/R editing on immature vs mature AMPAR (lines 117-120) and 2) highlight that any mechanism of isoform specific regulation is unlikely to occur through direct contact with the Q/R site itself (line 154).

*“Currently the logic is: protein A and protein B perhaps interact across this interface. To test this interaction, we will use mutation in A away from the interaction interface as it is known to be important for A.”*

We would like to clarify that our logic is the following (using similar language as the reviewer):

1) Protein A (AMPAR) and protein B (Ly6) could interact at the cell surface. As shown in Fig 1, the interaction interface is likely to be extracellular due to predicted anchoring of Ly6 proteins to the extracellular face of the membrane.
2) Prior work on other family members for protein B suggest roles in regulation of ionotropic receptor function. Prior work on Protein A (an ionotropic receptor) demonstrates multiple examples of regulation via extracellular interactions with small regulatory proteins.
3) Specifically, Protein A has different isoforms and combinations that each have unique biochemical properties and biological functions. One type of regulation is the effects of small regulatory proteins on properties of homomeric Protein A^Q^ and Protein A^R^.
4) As there has been no prior work investigating the possibility of interaction and/or regulation between Protein A and Protein B, we choose to begin our work by examining the simplest molecular version of Protein A that still has relevance for function: homomeric Protein A^Q^ and Protein A^R^.

Our hypothesis: If protein B is involved in regulation of function of protein A^Q^ and protein A^R^, then protein B may interact differently with protein A^Q^ and protein A^R^.

As noted in our previous revision, we found Reviewer #2’s comments very helpful in clarifying our hypothesis and we included substantial changes to our text to highlight that our hypothesis does not rule out the possibility of protein B interacting or regulating other forms of protein A. Given that there was no information available on regulation of protein A by protein B, we began with this hypothesis with these two isoforms as a simple starting point in our investigations (working with a homomeric receptor with only one amino acid change) that would still contribute knowledge to the field (working with isoforms that have been previously studied and have significant functional implications). Consequently, our choice to study the Q/R isoforms was *not* to “use the mutation to test the interaction interface,” but rather as a simpler system for evaluating potential effects on AMPAR function. While we understand there can be a variety of approaches towards investigating a completely uncharacterized system (here, Ly6 protein interaction with AMPAR), we hope this clarifies our approach to the reviewer. We have included additional language in lines 108-118 to clarify this in the text.

*“Why not find examples of compounds/peptides that interact with extracellular domains of AMPARs and differ between Q and R variants (I am not aware of any, but I might be wrong)?”*

To our knowledge, we are also unable to identify the reagents the reviewer is suggesting, and so would be unable to do this experiment.

*“Or perhaps there is a mutation in the pore region of AChRs that affects binding of alpha-bungarotoxin? If so, this would introduce well the rest of the paper.”*

To our knowledge there is not a mutation in the pore region of nAChRs that affects binding of bungarotoxin. We did find a work studying the influence of a pore mutation T288N on GABA_A_ receptor trafficking^5^; however, this was absent data related to any potential impacts on auxiliary and/or small regulatory protein function.

^5^Hernandez, C.C., Zhang, Y., Hu, N. *et al.* GABA **_A_** Receptor Coupling Junction and Pore *GABRB3* Mutations are Linked to Early-Onset Epileptic Encephalopathy. *Sci Rep* **7**, 15903 (2017). https://doi.org/10.1038/s41598-017-16010-3.

*"AMPARs have also been expressed as a trimeric complex in a heterologous system (A1 + y8 + CNIH-2 in HEK cells in Kato et al., Neuron, 2010;* [*https://doi.org/10.1016%2Fj.neuron.2010.11.026*](https://doi.org/10.1016%2Fj.neuron.2010.11.026)*; PMID: 21172611), so still not sure it makes sense to use it only as a pulldown control and not in an experiment which would substantially strengthen the paper (in my opinion).”*

We acknowledge and appreciate the reviewer’s opinion on this experiment, and have included the new citation in line 294. As we noted in our previously revised text, our experiments do not exclude the possibility of a trimeric complex and that is a source for future work.

*“1) Thank you for making it clear that n is 3 or more for all experiments, but it would be good to specify the exact number of replicates (n = x) for each experiment. Some quantification of at least some of the experiments would also strengthen the paper.”*

We have updated the specific n for each experiment in the figure legends. With regards to quantification—we are unsure as to which experiments the reviewer is referring to. For example, while we recognize the value in quantifying Western Blot intensities for certain experiments (for example, quantifying differences in surface expression for surface biotinylation or labeling experiments), given that our results did not show an interaction we are unsure what the benefit of quantifying those bands (or in our case, the lack of bands) would be.

*“2) Figure 1 – nice figure! Orienting the two peptides in the same way would help comparison and it would also make it clearer how similar the fold is.”*

Thank you. The convention for orienting structures of Ly6 proteins in the literature is generally with the disulfide bonds at the “top” of the image and the three loops facing “down;” we have maintained this orientation in panel B to maintain consistency with depictions in the field. To help the reader orient, we intentionally kept the color and representation of the disulfide bonds (gold sticks) consistent between Lynx1 in panel A and the model of lypd2 in panel B to facilitate comparison between the two proteins.

*“3) Line 104-105: "AMPA receptors are tetrameric and can exist as heteromeric combinations of GluR1 with either GluR2 or GluR3 or as homomers of either GluR1 or GluR2."

To me this reads like AMPARs can form A1/A3 heteromers. At least in vivo, much more dominant heterotetramer combinations are A1/A2/A1/A2, A3/A2/A3/A2 and A1/A2/A3/A2 (see e.g. Yu et al, Nature, 2021;* [*https://doi.org/10.1038/s41586-021-03540-0*](https://doi.org/10.1038/s41586-021-03540-0)*).”*

We thank the reviewer for their close reading of the text, and have adjusted the language in lines 104-105 to be clearer.

*“4) "Several auxiliary proteins, including TARPs (such as stargazin), GRIP2/ABP, PICK1, and CNIHs and stargazin, have already been identified as playing key roles in AMPAR trafficking to the cell surface (38–40)."

GRIP2 and PICK1 do interact with AMPARs, but are not considered to be their auxiliary subunits in the way TARPs (and others) are (Schwenk et al., Neuron, 2012).”*

We have adjusted the language in lines 76-78 to reflect this and included two additional citations (both the reviewer’s suggestion and the one below).

Bissen D, Foss F, Acker-Palmer A. AMPA receptors and their minions: auxiliary proteins in AMPA receptor trafficking. Cell Mol Life Sci. 2019 Jun;76(11):2133–69.
